# Interaction of piRNA-like sequences from the 3′-UTR of SARS-CoV-2 with mRNA regions

**DOI:** 10.1016/j.gendis.2023.01.012

**Published:** 2023-03-22

**Authors:** Laura Pérez-Campos Mayoral, María Teresa Hernández-Huerta, Carlos Romero Díaz, Carlos Alberto Matias-Cervantes, Eduardo Pérez-Campos Mayoral, Margarito Martínez Cruz, Juan de Dios Ruiz-Rosado, Edgar Gustavo Ramos Martínez, Marco Antonio Sánchez Medina, Eduardo Pérez-Campos

**Affiliations:** a Research Center, Faculty of Medicine UNAM-UABJO, Autonomous University “Benito Juárez" of Oaxaca (UABJO), Oaxaca 68020, Mexico; b CONACyT, Faculty of Medicine and Surgery. Autonomous University “Benito Juárez" of Oaxaca (UABJO), Oaxaca 68020, Mexico; c National Technology of Mexico (TecNM)/IT Oaxaca, Oaxaca de Juárez, Oaxaca 68030, Mexico; d The Research Institute at Nationwide Children's Hospital, Columbus 43205, USA; e Facultad de Química, Universidad Nacional Autónoma de México, Coyoacán 04510, Mexico

Piwi-interacting RNAs (piRNAs of 26–32 nt in length) regulate gene expression, epigenetics, and transcriptional and post-transcriptional processes.^1^ In humans, piRNAs are expressed in germline and somatic tissues, and generally have a uracil at the 5′-end (position +1), an adenine at the +10 position, and are 2′-O methylated at the 3′-end.^2^ Onice piRNAs form the RNA-induced silencing complex (RISC) with PIWI proteins, they serve as guides to find their complementary sequences of the target mRNA, thus initiating their degradation. The Wuhan patient's genome is approximately 265 nucleotides (nts) in its 3′-UTR; piRNAs-like sequences have been reported in this region.^3^ Our objective was to search, through bioinformatics, for mRNA sequences that were homologous to the reverse complementary of the previously reported piRNA-like sequences from the 3′-UTR of SARS-CoV-2,^3^ and identify the possible interaction between them.

Considering that piRNAs bind to their target sequence in a reverse complementary manner, we used the reported piRNA-like sequences as templates and translated them into their reverse complementary sequences: 29757–29784, 29792–29819, 29692–29719, 29667–29694, 29684–29711, 29841–29868, and 29828–29855. We searched for homologous mRNA sequences in NCBI using BLASTN with the following criteria: i) eukaryotic cell gene mRNA and ii) at least 10 nt of identity to the reverse complementary sequences. Using the Nucleic Acid Builder (NAB) from AMBERTOOLS 20 (https://ambermd.org/), we designed the three-dimensional structures of piRNA-like sequences of the 3′-UTR of SARS-CoV-2 and its corresponding reverse complementary homologs (5′ to 3′ single-stranded). We also designed a double-stranded structure (sequence 29757–29784) with its reverse complementary sequence (cr-29784-29757), used as a control. The 3D structures in PDB format were docked with HEX 6.3, taking care that the resulting complexes were oriented 5′–3′ for receptors and 3′–5′ for ligands. Using the coupled structures from the previous stage with the best orientation, 26 ns (ns) of molecular dynamics (MD) were performed using AMBER 20 cuda code. Visualization of trajectories was performed using VMD 1.9.3. The distances of the coupled structures were calculated with RasMol for Linux. The pucker conformations, RMDS, and the distances between the nts of the trajectory were calculated by cpptraj. Figure editing was done with Chimera 1.16. The 29757-29778-vs-NCOA1 and 29828-29855-vs-IGSF9B complexes interacted stably. The distances between N3–N1 and O4–N6 that form H-Bonds with U-A, as well as O2–N2, N3–N1, and N4–O6 were conserved along the trajectory, unlike the rest.

We found eight sequences in the mRNA of NCOA1 (NG_029014.2, nts 178, 123 to 178, 150), RB1 (NM_00123.3, nts 3492 to 3519), RGL3 (NM_00116161.3, nts 2021 to 2048), AGMO (XM_011515402.4, nts 2048 to 2075), CAMK4 (NM_001323375.2, nts 5021 to 5044), CAMK4 (NM_001323375.2, nts 5024 to 5050), ELOA (NM_003198.3, nts 91 to 21), and IGSF9B (NM_001277285.4 nts 10115 to 10142) that were homologs to the reverse complementary of the piRNA-like sequences present in the 3′-UTR of SARS-CoV-2 (Table S1).

Couplings with HEX 6.3 showed an interaction between the piRNA-like sequences, 29757–29784 and 29828–29855, against their homolog to reverse complementary sequences (nts of the NCOA1 and IGSF9B mRNAs), with affinity energies of −981 kcal/mol and −1356 kcal/mol, respectively. In addition, we observed complementarity since the orientation of the receptors was 5′–3′ and that of the ligand was 3′–5' (Fig. 1A). The MD of the sequence 29757–29784 and its complementary reverse sequence cr-29784-29757 (control) remained stable, with an average RMSD of 6.19 ± 1.13 Å. Similarly, the trajectories of structures 29757–29784 *vs*. NCOA1 and 29828–29855 *vs*. IGSF9B presented average/mean RMSDs of 4.31 ± 0.79 and 4.73 ± 0.93 Å, respectively (Fig. 1B). Using cpptraj (https://amberhub.chpc.utah.edu/cpptraj/), we calculated the distances between the atoms that define the stability of the helical structures and verified that the control, structures 29757-29784-vs-NCOA1 and 29828-29855-vs-IGSF9B, remained stable (Fig. 1C and Table S2; Video S). Also, the pucker conformations for nucleotides 1–5, 12–16, and 24–28 were calculated for complexes 29757-29778-vs-cr-29778-29757, 29757-29778-vs-NCOA1, and 29828-29850-vs-IGSF9B (Fig. 1D). The calculation of distances in our models, both in the couplings using HEX 6.3 and the distances calculated along the MD trajectory of 29757-29784-NCOA1 and 29828-29855-IGSF9B, are stable when compared to the Watson-Crick model, based on the work of Seeman et al.^4^

**Figure 1:**
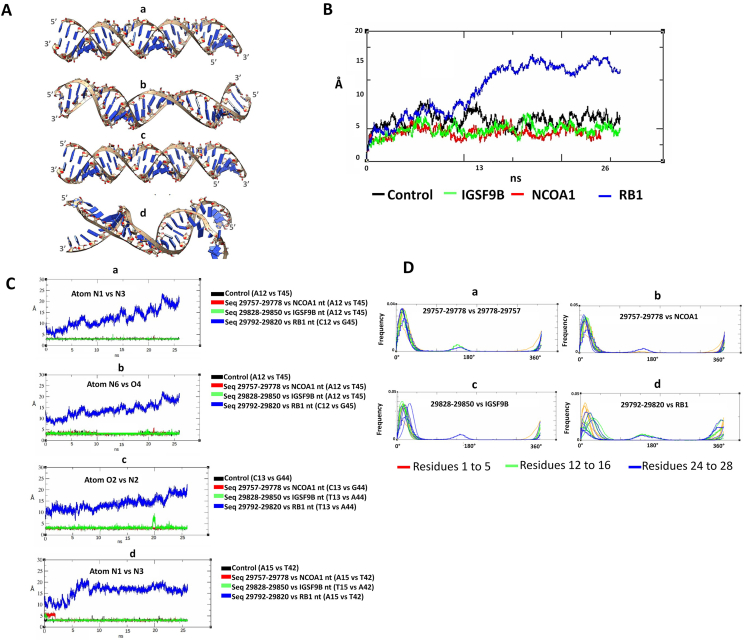
piRNA-like sequences from the 3′-UTR of SARS-CoV-2 with mRNA regions. **(A)** 3D structures of the control and RB1 complexes. **(a, b)** Control complex at 0 and 26 ns, respectively. The helix is shown to remain conserved. **(c, d)** The seq-29792-29819-vs-RB1 complex is shown at 0 and 26 ns, respectively. These helixes tend to separate. **(B)** RMSD control trajectories; seq-29757-29778-vs-NCOA1, seq-29828-29850-vs-IGSF9B, and seq-29792-29819-vs-RB1 sequences. We observed that the control complexes, NCOA1 and IGSF9B, remain stable, unlike RB1 which tends to flow upwards. **(C)** Distances between atoms that form H-bonds: **(a)** Atom N1 *vs*. N3, **(b)** Atom N6 *vs*. O4, **(c)** Atom O2 *vs*. N2, and **(d)** Atom N1 *vs*. N3. All remained stable, unlike the distances between the atoms of the seq-29792-29819-vs-RB1 complex (blue line). **(D)** Pucker conformations for nucleotides 1 to 5, 12 to 16, and 24 to 28 of the complexes: **(a)** 29757-29778-vs-29778-29757, **(b)** 29757-29778-vs-NCOA1, and **(c)** 29828-29850-vs-IGSF9B. The angles of this conformation are on the order of 0–45°, corresponding to a C3′ endo conformation, characteristic of ribonucleic acids, and a lesser extent on the order of 345–360°. **(d)** 29792-29819-vs-RB1 complex. Angles are more widely distributed from 0 to 60° and from 345 to 360°, indicative of O4′ endo and C2′ exo conformation; this may be due to helix decay along the trajectory.

Supplementary video related to this article can be found at https://doi.org/10.1016/j.gendis.2023.01.012.

The following is the supplementary data related to this article:

Multimedia component 3 Multimedia component 3

Nuclear receptor coactivator 1 (NCOA1) serves as a coactivator of different nuclear steroid receptors (SR), retinoid receptors (RR), prostanoid receptors (PR), and thyroid hormone; in turn, initiates the transcriptional process of genes such as interleukins (IL-12p40). While IGSF9B encodes a 763 amino acid protein that contains a fibronectin-like type III domain, suggesting its involvement in cell signaling. IGSF9B is mainly present in the brain and is associated with psychiatric illnesses and seizures, Parkinson's disease, and breast cancer where its presence is associated with a best prognosis.^5^ Whether SARS-CoV-2 infection impacts these genes would be important to correlate with phenotype; however, both genes (NCOA1 and IGSF9B) have not yet been studied in COVID-19 patients, thus suggesting a new topic for further study.

Although these results have the limitation that they correspond to an *in-silico* analysis, they are novel. Therefore, they could be significant for the secondary manifestations of SARS-CoV-2 infections, such as in long COVID.

In summary, two piRNA-mRNA complexes were found, 29757-29784-NCOA1 and 29828-29855-IGSF9B, whose results of the homologous sequences of the complementary reverse sequences from the 3′-UTR of SARS-CoV-2 could serve as guides for the RISC and initiates the degradation process of the target mRNA. This finding must be confirmed experimentally.

## Funding

This work was supported by the Faculty of Medicine of the UABJO, Oaxaca, Mexico, and by the National Technology of Mexico (TecNM) and CONACYT (BP-PA-2021050723-4900732-959110).

## Conflict of interests

The authors declare no conﬂict of interests.
